# Direct evidence for distinct colour origins in ROY polymorphs†

**DOI:** 10.1039/d1sc04051k

**Published:** 2021-09-01

**Authors:** Lisette R. Warren, Evana McGowan, Margaret Renton, Carole A. Morrison, Nicholas P. Funnell

**Affiliations:** University of Edinburgh Joseph Black Building, David Brewster Road Edinburgh EH9 3FJ UK c.morrison@ed.ac.uk +44 (0)131 650 4725; ISIS Neutron and Muon Facility, Rutherford Appleton Laboratory Didcot OX11 0QX UK nick.funnell@stfc.ac.uk +44 (0)1235 445385

## Abstract

ROY is one of the most well-studied families of crystal structures owing to it being the most polymorphic organic material on record. The various red, orange, and yellow colours of its crystal structures are widely-believed to originate from molecular conformation, though the orange needle (ON) polymorph is thought to be an exception. We report high-pressure, single-crystal X-ray measurements which provide direct experimental evidence that the colour origin in ON is intermolecular, revealing that the molecule undergoes minimal deformation but still exhibits a pronounced, reversible, pale orange → dark red colour change between ambient pressure and 4.18 GPa. Our experimental data are rationalised with band structures, calculated using an accurate hybrid DFT approach, where we are able to account for the variation in colour for five polymorphs of ROY. We highlight the outlier behaviour of ON which shows marked π⋯π stacking interactions that are directly modified through application of pressure. Band structure calculations confirm these intermolecular interactions as the origin of the colour change.

## Abstract 2

Alternative colour origins in ROY polymorphs are conclusively determined for the first time, using high-pressure diffraction and hybrid DFT.

## Graphical abstract

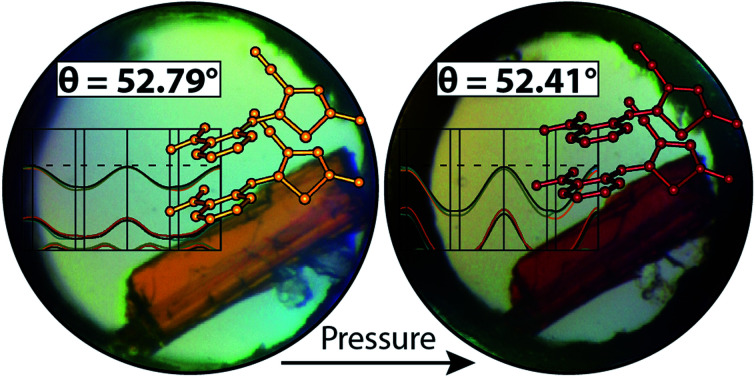

## 1. Introduction

**Fig. 1:**
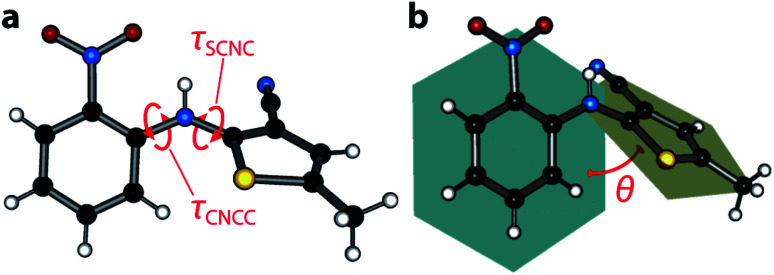
(a) Molecular structure of ROY. Carbon atoms are shown in black, nitrogen in blue, oxygen in red, sulfur in yellow, and hydrogen in white. The two rotatable bonds *τ*_SCNC_ and *τ*_CNCC_ that can influence intramolecular conjugation are indicated. (b) An alternate measure of the extent of ring coplanarity; the angle *θ* between mean planes calculated through the phenyl and thiophene groups.

The ‘ROY’ family of crystal structures, crystallising from the 5-methyl-2-[(2-nitrophenylamino)]-3-thiophenecarbonitrile compound, is numerous,^1–3^ with the most recent literature citing the discovery of the thirteenth form,^4^ making it the most polymorphic material in the Cambridge Structural Database (CSD) at the time of writing, ahead of other rivals including galunisertib (eight forms), the structurally-similar flufenamic acid (ten), and aripiprazole (twelve). The ROY molecule exhibits significant flexibility about the *τ*_SCNC_ dihedral angle between the crystal structures and, to a lesser extent, the *τ*_CNCC_ angle (shown in Fig. 1); the molecules show markedly different conformations and packing across all the known crystal forms.^5,6^ The propensity for ROY to form so many crystal structures spontaneously, from the melt or solution,^7^ has drawn significant attention from the crystal growth community, in attempts to control the polymorphic outcome.^8–13^ More recent work on crystal growth has exploited the cross-nucleating ability of ROY where synthetic analogues can be used to seed supercooled melts of structurally ‘normal’ material, leading to yet more polymorphs.^14^ The newest forms have been obtained *via* small-scale crystallisations from droplets of liquid ROY/oil-encapsulated ROY solutions.^4^

While ROY has been a fruitful system for experimentalists, with new polymorphs being found relatively frequently, calculating its solid state landscape and polymorph properties has proved more challenging. This is exemplified by the difficulties in ranking the internal energies of its many crystal structures—these often differ both from other theoretical studies and also from the experimentally-observed hierarchy.^15–18^ In some studies, some of the known forms of ROY have not ranked within the top one hundred most stable predicted structures at all.^16^ A complicating factor is the apparent difficulty in producing accurate potential energy surfaces as a function of molecular conformation; Thomas and Spackman have explored this in more detail, showing that DFT energies are unreliable for this purpose and are outperformed by MP2 calculations.^17^ Subsequently, Nyman *et al.* found that DFT+D approaches overestimate the stability of coplanar conformations, due to excessive predicted π-electron delocalisation.^18^ Though the ROY system is interesting in its own right, it also represents a more general challenge facing the materials chemistry community: how to compute accurate material physical properties when conventional DFT approaches—the ‘go-to’ method for solid-state calculations—prove too limited,^19^ in this case for a relatively simple geometry optimisation procedure.

The conformational flexibility of ROY in turn suggests a malleable electronic structure, and it is widely-accepted that its crystal colours arise from the degree of conjugation between the nitrophenyl and thiophene moieties.^6^ Each crystal form sits somewhere on the red–orange–yellow region of the visible spectrum (hence the ‘ROY’ moniker) where, broadly, coplanar and perpendicular molecular arrangements lead to red and yellow colours, respectively, and orange colours are represented by intermediate angles. Previous work by one of us demonstrated that the yellow (Y) polymorph exhibits piezochromic properties,^20^ where the crystal became progressively red in colour as the molecule was driven towards planarity, and crystal density increased, on applying pressure. Similarly, the molecular geometry of the orange plate (OP) form also showed susceptibility to pressure,^21^ though we were unable to comment on the crystal colour due to the lack of optical access to the pressure device.

Given the strong colouration of the ROY family, and its prominence in organic solid-state literature, there is a notable absence of calculated electronic band structures. A recent computational study by Feng *et al.*^22^ advanced this area, providing a set of band gap values for many of the ROY forms. However, the authors highlighted the aforementioned difficulties posed by DFT, which they note led to additional flattening (*ca.* 10°) of the *τ*_SCNC_ angle in the red and orange polymorphs, and systematic underestimation of the band gaps. A particularly intriguing observation, arising from their calculated molecular singlet excitation energies, was that the orange needle (ON) form appears to be an outlier; the authors postulated that its colour may actually originate from intermolecular interactions, in contradiction to the decades-held view that molecular conformation is predominantly responsible.

We have sought to explore the ROY colour origins in detail, reporting the first electronic band structures of ROY made possible by making use of a hybrid DFT approach with crystalline orbitals—its proven ability to calculate band gaps with greater accuracy than conventional DFT will likely see it gain further traction in the materials chemistry and physics communities,^23–25^ particularly in assisting band-structure engineering.^26,27^ In combination with direct evidence provided by high-pressure, single-crystal, X-ray diffraction measurements we show conclusively that the ON form is indeed an anomaly, where its colour is intermolecular in origin.

## 2. Results and discussion

### 2.1 High-pressure crystallography—cell compressibility

High-pressure diffraction provides the ideal experiment with which to test whether any observable change in colour occurs in conjunction with either intramolecular or intermolecular modifications (or both) to the structure. Earlier work on the Y form first revealed the piezochromic nature of ROY, showing that a reversible yellow → red colour progression was observed with pressure.^20^ This was accompanied by both a large deformation in the lattice—expected behaviour for a compressed molecular crystal structure—but also conformational change in the molecule. A similar structural response to pressure was also observed in the OP polymorph.^21^

**Fig. 2:**
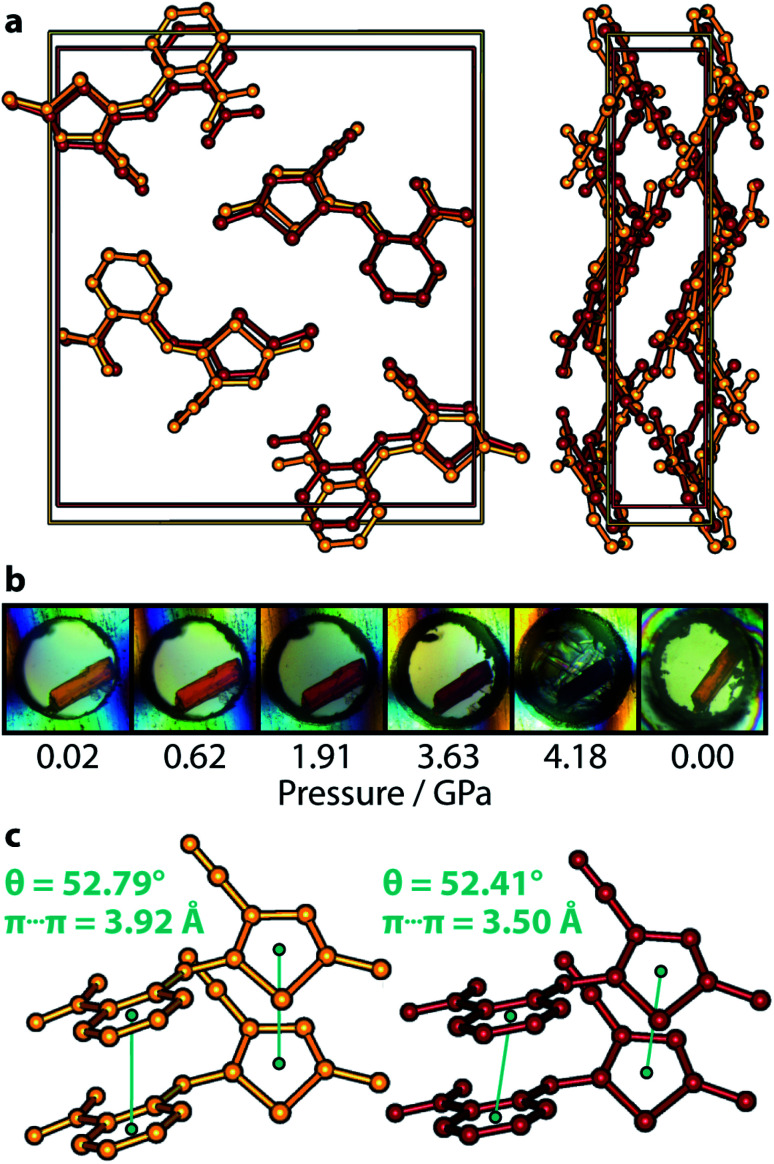
(a) ON form at 0.02 and 4.18 GPa, coloured orange and red, respectively. The left structure shows the view along the *a*-axis, right—view along the *c*-axis. (b) Colour progression in ON at select pressures. The dark red colour of the crystal is obscured at 4.18 GPa by crystallisation of the pressure medium. Decompression to ambient pressure shows complete reversibility of the colour change. (c) π⋯π-stacking motifs responsible for the colour change. The corresponding *θ* angle between mean planes shows negligible change between the lowest and highest-pressure structures. Hydrogen atoms in (a) and (c) are omitted for clarity.

Fig. 2 summarises the behaviour of ON ROY under pressure—there is no transformation in the crystal structure (see panel Fig. 2a); its *P*2_1_/*c* symmetry is retained over the pressure range investigated here. The unit cell simply becomes a more compressed version of its ambient-pressure form. Details of the cell compression characteristics are given more comprehensively in the ESI,† but we note here that the most compressible principal axis is approximately aligned with the *a*-direction, having a compressibility *K* of 21.6(7) TPa^−1^, corresponding to a decrease in length of 13.4%. The other two principal, orthogonal, directions are less compressible (11.5(5) and 3.7(10) TPa^−1^), ultimately leading to a bulk modulus *B*_0_ of 5.9(14) GPa. Table 1 provides crystal structure refinements statistics for select pressure points.

**Table 1:** Crystal refinement statistics for select pressure points; all data are available in the ESI. All structures have *P*2_1_/*c* symmetry with *Z* = 4, *Z*′ = 1. The relatively high *R*_1_ values are a consequence of both poor data quality (see low data completeness—reciprocal space coverage is restricted by the DAC body) and isotropic refinement of all C, N, and O atoms in order to maintain a favourable (>1 : 10) data : parameter ratio

Pressure/GPa	0.02	0.62	1.91	2.41	3.53	4.18	0.00
Chemical formula	C_12_H_9_N_3_O_2_S	C_12_H_9_N_3_O_2_S	C_12_H_9_N_3_O_2_S	C_12_H_9_N_3_O_2_S	C_12_H_9_N_3_O_2_S	C_12_H_9_N_3_O_2_S	C_12_H_9_N_3_O_2_S
Formula weight/g mol^−1^	259.29	259.29	259.29	259.29	259.29	259.29	259.29
Crystal system	Monoclinic	Monoclinic	Monoclinic	Monoclinic	Monoclinic	Monoclinic	Monoclinic
Space group	*P*2_1_/*c*	*P*2_1_/*c*	*P*2_1_/*c*	*P*2_1_/*c*	*P*2_1_/*c*	*P*2_1_/*c*	*P*2_1_/*c*
*a*-Axis/Å	3.920050(15)	3.767380(17)	3.631200(17)	3.59640(2)	3.52400(2)	3.49690(2)	3.94747(2)
*b*-Axis/Å	18.56291(5)	18.14088(5)	17.68860(4)	17.60380(6)	17.38790(7)	17.29910(6)	18.67316(6)
*c*-Axis/Å	16.40883(5)	16.22092(5)	16.02610(5)	15.95340(6)	15.81840(7)	15.77880(6)	16.33187
*β*/°	93.655(6)	91.918(5)	89.648(5)	89.358(7)	88.361	88.037(7)	93.736(7)
Volume/Å^3^	1191.601(11)	1107.975(8)	1029.350(6)	1009.951(8)	968.875(10)	953.950(8)	1201.293(13)
Density/g cm^−3^	1.445	1.554	1.673	1.705	1.777	1.805	1.434
Parameters	82	82	82	82	82	82	82
Unique reflections	1191	1095	984	956	925	880	1188
*R*_1_(*I*/*σ* > 2.0)	9.51	8.09	11.52	11.39	8.49	8.13	5.49
Goodness of fit	0.91	1.06	1.08	1.01	1.08	1.15	0.96
Δ*ρ*_max_, Δ*ρ*_min_/e Å^−3^	−1.09, 0.99	−0.85, 0.90	−0.86, 0.80	−0.77, 0.74	−0.83, 0.95	−0.91, 0.82	−0.77, 0.80
Completeness (*d*_min_ = 0.90 Å)	69.9%	69.3%	69.1%	68.8%	67.9%	65.5%	69.2%

### 2.2 High-pressure crystallography—colour origin

Similar to the Y form, there is a clear pale orange → red colour progression as pressure is increased (Fig. 2b). This is completely reversible, with the crystal returning to a pale orange colour on complete decompression. The deep red colour of the crystal is obscured at 4.18 GPa, due to the apparent crystallisation of the pentane mixture at a lower-than-expected pressure (ordinarily 5.4 GPa); premature crystallisation of the *n*-pentane component is now known to occur on occasion.^28^

Fig. 2c depicts the most conclusive experimental observation that the crystal colour arises from intermolecular interactions; the conformational geometry of the molecule is insensitive to the effects of pressure, showing only negligible change. Though the molecular conformation is usually discussed in terms of the *τ*_SCNC_ angle, which measures 52.9(11)° at 4.18 GPa (*cf.* 52.6(3)° at ambient pressure,^29^ and 47.7(13)° at 0.02 GPa), a more accurate measure of the conformation is the angle made between mean planes *θ* drawn through the phenyl and thiophene groups, shown schematically in Fig. 1b as this does not neglect the (often small) effect of the *τ*_CCNC_ dihedral angle. Between 0.02 GPa and 4.18 GPa, *θ* is effectively unchanged, decreasing from 52.79 to 52.41°, and measures 53.68° for the literature 0 GPa structure (CSD ref. code: QAXMEH). As an aside, we note that the difference between *τ*_SCNC_ and *θ* can actually be quite pronounced in some other ROY forms, *e.g.* for the R form (QAXMEH02), they measure 21.74° and 45.56°, respectively.

Lastly, the relatively compressible *a*-axis indicates that the most deformable intermolecular interactions are aligned with this direction. These are comprised of two sets of π⋯π stacking interactions, one between nitrophenyl rings and the other between thiophene groups. The crystal symmetry constrains both of these interactions to be the same length, which decrease from 3.92 Å at 0.02 GPa to 3.50 Å at 4.18 GPa. The authors of ref. 22 postulated that intermolecular interactions are most likely to be responsible for the colour origin in ON are the π⋯π stacks—our experimental results show that this is almost certainly the case.

### 2.3 Electronic band structures

In order to more fully understand the colour origin in ROY polymorphs—specifically, the nature of the orbitals involved—we performed electronic structure calculations using a hybrid DFT approach that has a proven track record in determining band gaps.^23–25^ Five polymorphs (red R, orange plate OP, orange needle ON, yellow Y, and yellow needle YN) that encompass the full colour spectrum exhibited by ROY were selected for computation—starting coordinates for each were obtained from literature structures in the CSD. First, geometries were optimised, while holding unit cell parameters fixed at experimental values. A strong level of agreement between our calculated interplanar angles *θ* and the experimentally observed angles, shown in Table 2 confirms that the simulations are providing accurate models of the original crystal structures, avoiding the over-stabilisation of the more planar forms seen with conventional DFT.^18^

**Table 2:** Comparison of optimised and experimental molecular geometries. Both the calculated *τ*_SCNC_ and *θ* angles show good agreement with experimental values, corroborated by low root mean square deviation (RMSD) values calculated between the pairs of structures in Mercury CSD. The calculated band gap is given for each polymorph

Polymorph	R	OP	ON	YN	Y
*τ*_SCNC_ (expt.)/°	21.7	46.1	52.6	104.0	104.7
*τ*_SCNC_ (calc.)/°	20.9	46.6	53.4	105.6	108.6
*θ* (expt.)/°	45.6	50.7	53.7	109.1	106.2
*θ* (calc.)/°	44.7	52.2	54.7	109.7	109.5
RMSD/Å	0.041	0.099	0.118	0.056	0.063
Band gap/eV	1.95	2.37	2.32	2.54	2.80

**Fig. 3:**
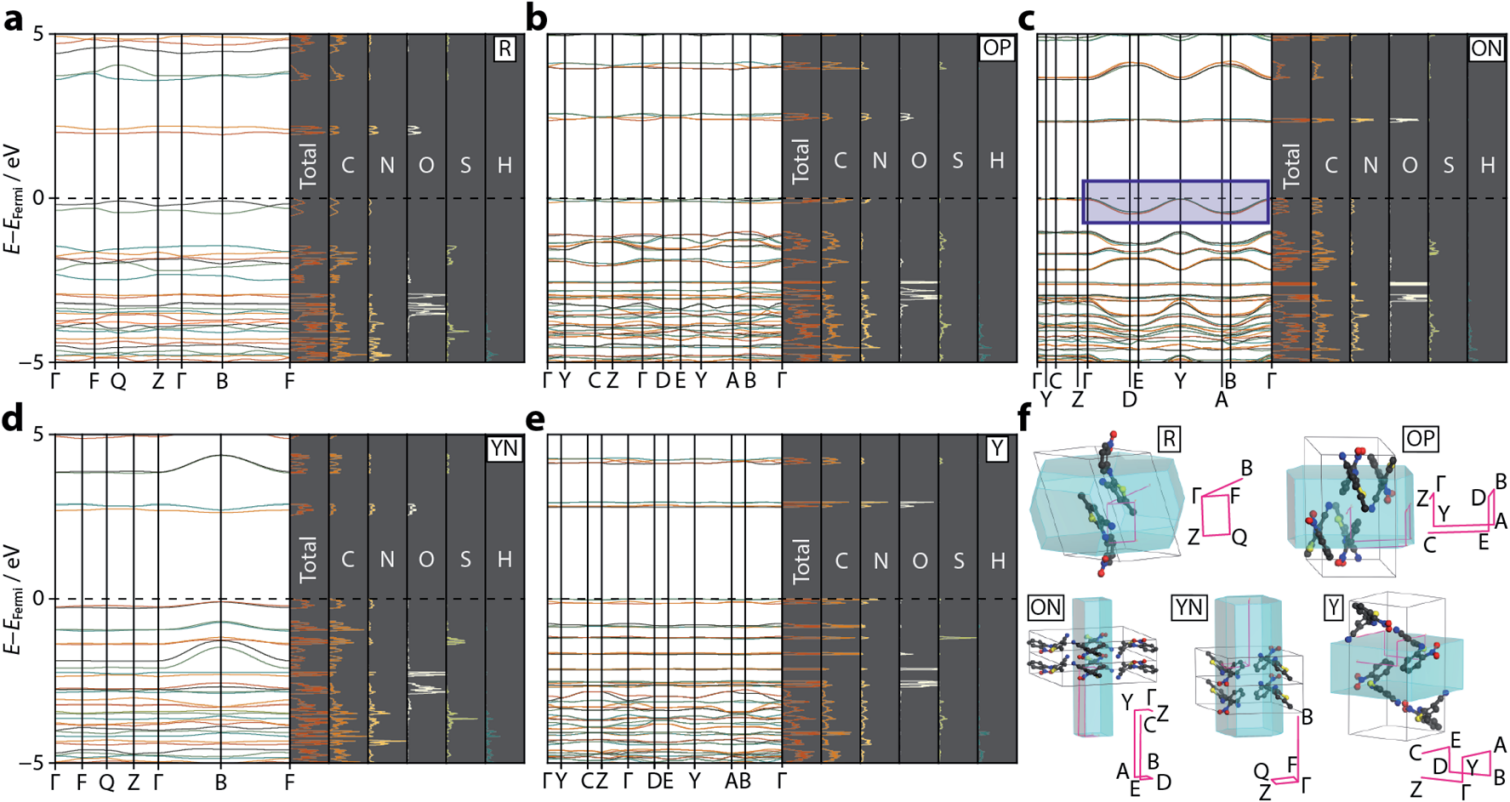
Calculated electronic band structure diagrams for the ROY polymorphs: (a) R, (b) OP, (c) ON, (d) YN, and (e) Y. On each panel the left hand side shows the band dispersion for the relevant *k*-point paths through the Brillouin zone. The right hand, shaded, panels show the total PDOS, along with the individual atom contributions. The dotted black line shows the Fermi level, set to 0 eV in each case (absolute *E*_Fermi_ values are: R = −5.49, OP = −5.80, ON = −5.73, YN = −6.00, Y = −6.04 eV). The blue rectangular region in (c) highlights the anomalous energy dispersion in the valence bands for ON. (f) A visualisation of the Brillouin zone paths (pink) in each polymorph, compared with the real-space unit cells. Each sampled *k*-point is labelled. Hydrogen atoms are omitted for clarity.

Following optimisation, electronic band structure diagrams and projected density of states (PDOS) were computed, with the outputs shown in Fig. 3 alongside their respective Brillouin zone paths. The computed band gap values are given in Table 2 and appear to correlate well with the known colours of the polymorphs, providing some confirmation that the hybrid DFT approach does not suffer from the same difficulties as conventional DFT, in agreement with observations made in ref. 22. For R, the computed band gap (1.95 eV) is similar to the experimental value reported for α-HgS (2.0 eV), from which the red pigment vermillion is derived.^30^ The orange polymorphs ON and OP present near-identical band gaps of 2.32 and 2.37 eV, respectively, which closely matches that of the orange pigment lead(ii) chromate (2.3 eV),^31^ while the larger band gaps for YN and Y (2.54 and 2.80 eV) are similar to CdS (2.5 eV), which is used to obtain the pigment cadmium yellow.^32^

The strong link between observed colour and electronic band gap confirms that the electronic transitions responsible for the colouration of ROY are confined to the frontier orbitals, located either side of the Fermi energy level. From the band structure plots—shown in Fig. 3—we observe two valence and two conduction bands for R and YN, whereas ON, OP, and Y have double this number; this simply reflects the number of molecules in each respective unit cell. The frontier bands are largely *k*-invariant, indicating that the corresponding crystalline orbitals are localised at the molecular level and are not strongly influenced by the lattice environment. However, as identified in ref. 22, ON is an exception (Fig. 3) as the valence bands show a degree of energy dispersion with respect to the *k*-point paths Γ → D/B and Y → A/E. Superposition of the Brillouin zone path with the real-space crystal lattice (Fig. 3f) shows that this corresponds to the *a*-direction, suggesting that the pertinent intermolecular interactions must be aligned with this unit cell vector; notably the deformable π⋯π nitrophenyl and thiophene stacking interactions are coincident with this direction. However, the ring separation distance at ambient pressure is long (3.95 Å), so this interaction is likely to be weak.

### 2.4 Pressure-dependent band dispersion

**Fig. 4:**
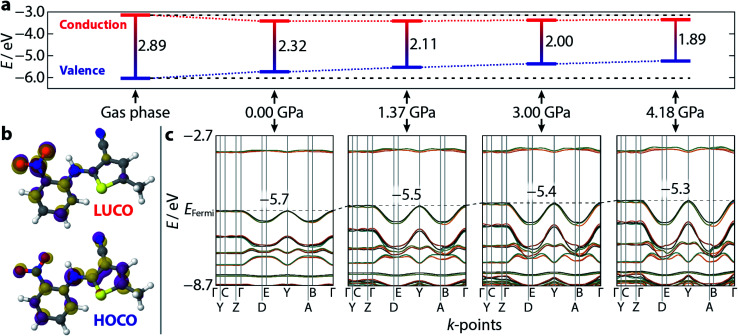
Calculated electronic band structure and orbital diagrams for ON–ROY as a function of pressure. (a) The calculated *Γ*-point band gap, at each pressure, and the absolute values of the valence (blue) and conduction (red) bands. A gas phase (isolated molecule) calculation is shown for reference. The horizontal black dotted lines are extrapolated from the gas phase structure for reference. (b) A visualisation of the lowest unoccupied, and highest occupied, crystalline orbitals (LUCO/HOCO) at ambient pressure—the isosurface value is 0.05 eV Å^−2^. The purple/gold colours of each individual orbital simply indicate opposite signs of the wavefunction. (c) The band structure for each crystalline model, corresponding to the band gaps shown in (a). The increasing extent of band dispersion with applied pressure is particularly evident at the top of the valence band. The black dotted line indicates the energy of the Fermi level (positioned at the centre of the *y*-axis at 0.0 GPa), which increases with pressure—its value is shown, inset, on each plot.

**Fig. 5:**
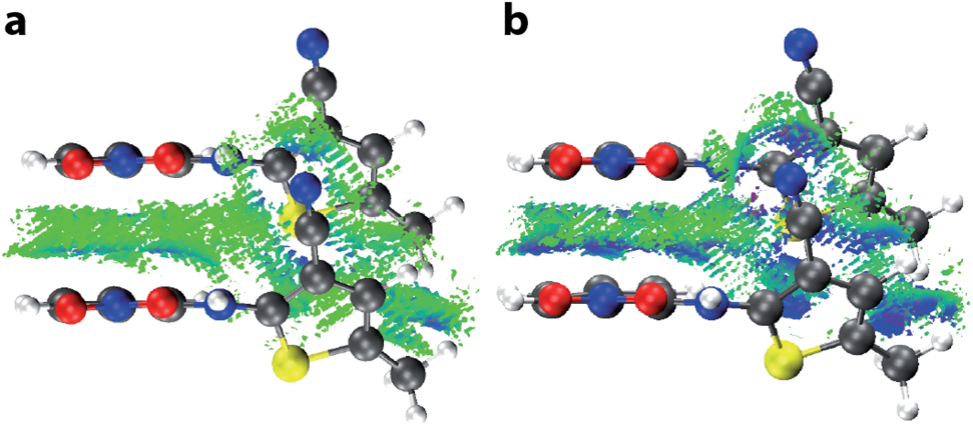
Non-covalent bonding interactions, represented by 3D isosurfaces (*s* = 0.5 a.u., −0.004 (blue) < *ρ* < 0 (green) a.u.). Interactions are shown for π⋯π-stacked ON–ROY molecules at (a) ambient pressure, and (b) 4.18 GPa.

Having demonstrated the reliability of our calculations in reproducing ambient-pressure molecular geometry and band gaps, we applied the same computational strategy to select high-pressure ON structures (1.37, 3.00, and 4.18 GPa), effectively accounting for the change in cell volume, which is known to have computational implications.^22^ These geometry optimisations returned *θ* values of 52.0, 51.6, and 51.1°, confirming that the molecular geometry is largely unaltered with pressure. The resulting electronic band structure diagrams are shown in Fig. 4, along with the ambient pressure band structure for direct comparison. It is immediately apparent the degree of dispersion in the frontier bands increases with applied pressure. The variance in energy is exclusively confined to the valence bands along the *k*-point paths Γ → D/B and Y → A/E. This implies that the strength of the intermolecular interactions along the *a*-direction increase with applied pressure; an observation substantiated by a contraction in the aforementioned π⋯π stacking of 0.45 Å, at the highest pressure measured here. Non-covalent interaction (NCI) plots for the optimised structures at ambient pressure and 4.18 GPa, shown in Fig. 5 show the reduced electron density gradient isosurface (*s*), coloured according to the strength (*ρ*) of the interactions. The enhanced blue colouration in the high-pressure structure indicates stronger π⋯π stacking interactions—approximately double that of the ambient pressure structure. Crucially, the simulations support the presence of piezochromic behaviour as the size of the band gap reduces from 2.32 to 1.89 eV as pressure is applied, corroborating the pale orange → red colour change that is visually evident in Fig. 2.

### 2.5 Frontier crystalline orbitals

Plotting the values of the valence and conduction band energies raises a further interesting point, namely that the pressure-induced decrease in band gap can be attributed, almost entirely, to an increase in energy of the valence band—see Fig. 4. This in turn can be accounted for by the dispersion in these frontier bands, which was explained on the basis of the π⋯π stacking interactions. Fig. 4 also shows the frontier orbital energy separation for an isolated (gas phase) molecule of ROY with atomic coordinates frozen to those obtained from geometry optimisation of the ambient pressure structure. The influence of the crystalline environment clearly impacts on both valence and conduction band energies, serving to raise the former and reduce the latter.

More specific information on the nature of these frontier bands can be provided by the PDOS, shown in Fig. 3 alongside the ambient-pressure band structure diagrams. From these, we can deduce that the top of the valence band is derived mostly from carbon and nitrogen states, with a small contribution from oxygen, whereas the bottom of the conduction band has near equal weighting across all three atomic states. Sulfur makes a small contribution to both orbital states. This behaviour is mirrored across all five polymorphs investigated here, suggesting that the frontier orbitals are invariant to crystal packing.

Fig. 4 also presents a visualisation of the frontier orbitals at the *Γ*-point for the ON form. Both the highest occupied crystalline orbital (HOCO) and lowest unoccupied crystalline orbital (LUCO) are π-type orbitals, with the HOCO delocalised across the whole molecule, while the LUCO is more localised on the nitrophenyl ring. Absorption of light will therefore likely accumulate electron density in the π-orbitals of the nitrophenyl region of the molecule, further enhancing the π⋯π interactions.

## 3. Conclusions

We have provided conclusive experimental and computational evidence that the colour origin in the ON ROY polymorph arises from intermolecular π⋯π interactions, making it something of an anomaly in the ROY family. These weak, deformable, interactions strengthen on application of pressure and, in doing so, introduce energy dispersion in the valence bands which progressively narrow the band gap to values commensurate with the colours seen experimentally. Though the colours in the other ROY polymorphs investigated here are clearly intramolecular in nature, the extended crystal lattice is still important as it plays a role in stabilising the respective molecular conformations. That ROY can adopt distinct mechanisms in different polymorphs to produce its strong colouration, is owed to its flexibility, and this has some precedent in other flexible polymorphic compounds.^33^ This only becomes evident by directly comparing the band structures between polymorphs—were the ON band structure calculated in isolation, the extent of inter/intramolecular influence would be less clear. The accuracy in geometry optimisation and band gap calculation of the hybrid DFT approach we have used is highly encouraging, and can be straightforwardly transferred to other solid-state materials. In particular, it has allowed us to ascertain the level of influence the intermolecular interactions have on a material property (in this case colour), through the extent of band dispersion.

Our study concerns just five of the thirteen known ROY polymorphs (and an additional three were also considered by Feng *et al.*),^22^ which leaves open the possibility that the colours of some of the other forms could also be a result of intermolecular excitations. The ROY polymorphs have been loosely categorised based on distinct regions they occupy on their conformational potential energy surface,^5,6^ however if additional polymorphs were revealed to show similar electronic behaviour to the ON form, then perhaps grouping the forms by intra/intermolecular colour origins might be an appropriate, alternative, classification system.

## 4. Methods

### 4.1 High-pressure X-ray diffraction

ROY was obtained in powdered form from TCI Chemicals as the OP polymorph. Small crystals of the ON form were visually identified, and isolated, from other concomitantly-occurring forms following recrystallisation from acetone; these were then used to seed saturated ROY:acetone solutions. A suitable crystal was identified and loaded in a Merrill–Bassett diamond anvil cell (DAC),^34^ equipped with Boehler–Almax anvils with an 85° opening angle and WC backing seats.^35^ A 1 : 1 volume mixture of pentane : isopentane was included as a pressure-transmitting medium,^36^ and a ruby chip as a pressure calibrant; pressure was determined using the ruby fluorescence method.^37^ X-ray diffraction data were collected on a Rigaku Synergy diffractometer, using Mo K_α_ radiation, at pressures of 0.02, 0.62, 1.37, 1.91, 2.41, 3.00, 3.53, and 4.18 GPa as well as a further measurement on complete decompression. The raw diffraction data were integrated and corrected for absorption using CrysAlisPro.

Structure refinements were carried out using Crystals.^38^ A starting model for the lowest-pressure refinement was obtained from an earlier ambient-pressure dataset (unreported). To avoid any potential bias of the dihedral angles, after atomic coordinates were imported, the entire thiophene moiety was deleted and relocated in a Fourier difference map. Owing to the low completeness of the data, only the sulfur atom was refined anisotropically, and hydrogen atoms were constrained to ride on their host atoms. All covalent bond distances were restrained to values informed by the ambient pressure structure, and 1–2, 1–3 vibration and thermal similarity restraints were also applied. Refined models at each pressure were then used as a starting set of coordinates for the following pressure point.

### 4.2 Hybrid DFT calculations

Solid-state calculations were performed using CRYSTAL17,^39,40^ with triple zeta quality all-electron basis sets with valence polarisation used for all atoms, combined with the HSE06 hybrid functional, along with a Grimme D3 dispersion correction.^41,42^ This choice of functional can be justified from its proven track record in accurately calculating electronic band gaps.^23–25^ Ambient-pressure structures deposited in the CSD, and our high-pressure X-ray structure determinations of the ON form were used as input geometries for atom-only optimisation. *K*-Space was sampled using a Monkhorst–Pack net of 8 × 8 × 8 for all structures.^43^ Increasing the *k*-point sampling to a larger Monkhorst–Pack net of 16 × 16 × 16 proved convergence with respect to *k*-points had been achieved to within 1 × 10^−7^ a.u. Tolerances for the bielectronic Coulomb and exchange contributions to the Fock matrix are controlled by five parameters, the first four of which were set to 1 × 10^−7^, and the fifth to 1 × 10^−14^.^39,40^ Convergence criteria were set on the root-mean-square (RMS) and absolute values for both the gradient (*i.e.* atomic forces) and estimated atomic displacements at 3 × 10^−4^ a.u. and 1.2 × 10^−3^ a.u., respectively. In addition, the energy convergence threshold between successive cycles was required to be below 10^−7^ a.u.^39^

Following optimisation, the electronic band structures, PDOS and localised crystalline orbitals were obtained, with the latter computed at the Brillouin zone *Γ*-point. The non-covalent interaction plots were obtained using the CRITIC 2 code.^44–46^ For the isolated molecule optimisations, the same procedure as documented above was employed, with the exception that the contents of the unit cell were deleted to leave just one ROY molecule inside a non-periodic system, and the dihedral angle was constrained to the same value as observed in the ON crystal structure (53.4°).

## Data availability

Crystallographic and computational details/structures are provided in the ESI.†

## Author contributions

N. P. F. and C. A. M. conceived the study. L. R. W., E. M., M. R., and C. A. M. performed and analysed the band structure calculations. N. P. F. performed and analysed the high-pressure X-ray measurements. N. P. F. and C. A. M. wrote the manuscript.

## Conflicts of interest

There are no conflicts to declare.

## Supplementary Material

SC-012-D1SC04051K-s001

SC-012-D1SC04051K-s002

SC-012-D1SC04051K-s003

SC-012-D1SC04051K-s004

SC-012-D1SC04051K-s005

SC-012-D1SC04051K-s006

SC-012-D1SC04051K-s007

SC-012-D1SC04051K-s008

SC-012-D1SC04051K-s009

SC-012-D1SC04051K-s010
