# Supplementary material for: Direct evidence for distinct colour origins in ROY polymorphs
Source: Chem Sci. 2021 Sep 1;12(38):12711–8. doi: 10.1039/d1sc04051k (PMC8494124; doi:10.1039/d1sc04051k)
Supplement: SC-012-D1SC04051K-s009 [file SC-012-D1SC04051K-s009.pdf]

Electronic Supplementary Material (ESI) for Chemical Science.
This journal is © The Royal Society of Chemistry 2021

Direct evidence for distinct colour origins in ROY polymorphs

SUPPLEMENTARY INFORMATION

Lisette R. Warren,[a] Evana McGowan,[a] Margaret Renton,[a]
Carole A. Morrison[a,*] and Nicholas P. Funnell[b,*]

[a] University of Edinburgh, Joseph Black Building, David Brewster Road, Edinburgh, EH9 3FJ, U.K.

[b] ISIS Neutron and Muon Facility, Rutherford Appleton Laboratory, Didcot, OX11 0QX, U.K.

[*]To whom correspondence should be addressed;

E-mail: nick.funnell@stfc.ac.uk, c.morrison@ed.ac.uk

Contents

1 Crystal structure refinement statistics 3

2 Unit cell compressibility 5

3 High-pressure conformational change 6

4 ROY—conformational potential energy surface 7

5 References 8

1 Crystal structure refinement statistics

Table S1: Crystal structure refinement statistics for all pressure points. Note the relatively high $R1$ values are a consequence of both incomplete, poor-quality data (scattering through a diamond anvil cell) and only isotropic refinement of C, N, and O atoms. The latter point is due to the need to maintain a favourable data:parameter ratio.

Pressure / GPa	0.02	0.62	1.37	1.91	2.41
Chemical formula	$C_{12}H_9N_3O_2S$	$C_{12}H_9N_3O_2S$	$C_{12}H_9N_3O_2S$	$C_{12}H_9N_3O_2S$	$C_{12}H_9N_3O_2S$
Formula weight / g mol^{-1}	259.29	259.29	259.29	259.29	259.29
Crystal system	Monoclinic	Monoclinic	Monoclinic	Monoclinic	Monoclinic
Space group	$P2_1/c$	$P2_1/c$	$P2_1/c$	$P2_1/c$	$P2_1/c$
a-axis / Å	3.920050(15)	3.767380(17)	3.68945(2)	3.631200(17)	3.59640(2)
b-axis / Å	18.56291(5)	18.14088(5)	17.85212(5)	17.68860(4)	17.60380(6)
c-axis / Å	16.40883(5)	16.22092(5)	16.13731(5)	16.02610(5)	15.95340(6)
β / °	93.655(6)	91.918(5)	90.452(6)	89.648(5)	89.358(7)
Volume / Å^3	1191.601(11)	1107.975(8)	1062.843	1029.350(6)	1009.951(8)
Density / gcm^{-3}	1.445	1.554	1.620	1.673	1.705
Parameters	82	82	82	82	82
Unique reflections	1191	1095	1021	984	956
$R1(I/\sigma > 2.0)$	9.51	8.09	12.14	11.52	11.39
Goodness of fit	0.91	1.06	0.99	1.08	1.01
$\Delta\rho_{\max}$, $\Delta\rho_{\min}$ / e Å^{-3}	−1.09, 0.99	−0.85, 0.90	−0.93, 1.09	−0.86, 0.80	−0.77, 0.74
Completeness ($d_{\min}$ = 0.90 Å)	69.9%	69.3%	69.0%	69.1%	68.8%

Table S2: Crystal structure refinement statistics—continued

Pressure / GPa	3.00	3.53	4.18	0.00
Chemical formula	$C_{12}H_9N_3O_2S$	$C_{12}H_9N_3O_2S$	$C_{12}H_9N_3O_2S$	$C_{12}H_9N_3O_2S$
Formula weight / g mol^{-1}	259.29	259.29	259.29	259.29
Crystal system	Monoclinic	Monoclinic	Monoclinic	Monoclinic
Space group	$P2_1/c$	$P2_1/c$	$P2_1/c$	$P2_1/c$
a-axis / Å	3.565200(17)	3.52400(2)	3.49690(2)	3.94747(2)
b-axis / Å	17.43660(5)	17.38790(7)	17.29910(6)	18.67316(6)
c-axis / Å	15.97730(5)	15.81840(7)	15.77880(6)	16.33187
β / °	88.803(6)	88.361	88.037(7)	93.736(7)
Volume / Å^3	993.012	968.875(10)	953.950(8)	1201.293(13)
Density / gcm^{-3}	1.734	1.777	1.805	1.434
Parameters	82	82	82	82
Unique reflections	876	925	880	1188
$R1(I/\sigma > 2.0)$	8.88	8.49	8.13	5.49
Goodness of fit	1.07	1.08	1.15	0.96
$\Delta\rho_{\mathrm{max}}$, $\Delta\rho_{\mathrm{min}}$ / e Å^{-3}	−0.78, 0.61	−0.83, 0.95	−0.91, 0.82	−0.77, 0.80
Completeness (d_{min} = 0.90 Å)	63.8%	67.9%	65.5%	69.2%

2 Unit cell compressibility

The ROY ON unit cell is monoclinic, and so the *a*- and *c*-axes are not constrained to orthogonality. In order to properly understand the compressibility characteristics of the cell, it is necessary to reformulate the cell vectors on an orthogonal axis system, where each axis is aligned with a principal direction of the strain tensor. The principal directions are defined as $\mathbf{X_1} \simeq -\mathbf{a} - 0.1\mathbf{c}$, $\mathbf{X_2} = \mathbf{b}$, $\mathbf{X_3} \simeq -0.9\mathbf{a} - 0.4\mathbf{c}$. Using PASCal,[S1] we calculate the compressibility of each direction $\mathbf{X_1} = 21.6(7)$, $\mathbf{X_2} = 11.5(5)$, and $\mathbf{X_3} = 3.7(10)$ TPa^{-1}, as well as the bulk modulus using a third-order Birch–Murnaghan fit ($B_0 = 5.9(14)$ GPa, $B' = 12(3)$, $V_0 = 1196(11)$ Å^3.[S2] The relatively large errors on the bulk modulus parameters reflect a less–than–ideal fit to the volume data. A second-order Birch–Murnaghan fit was completely unable to describe the form of the pressure–volume curve. A visual summary of these quantities is given in Figure S1.

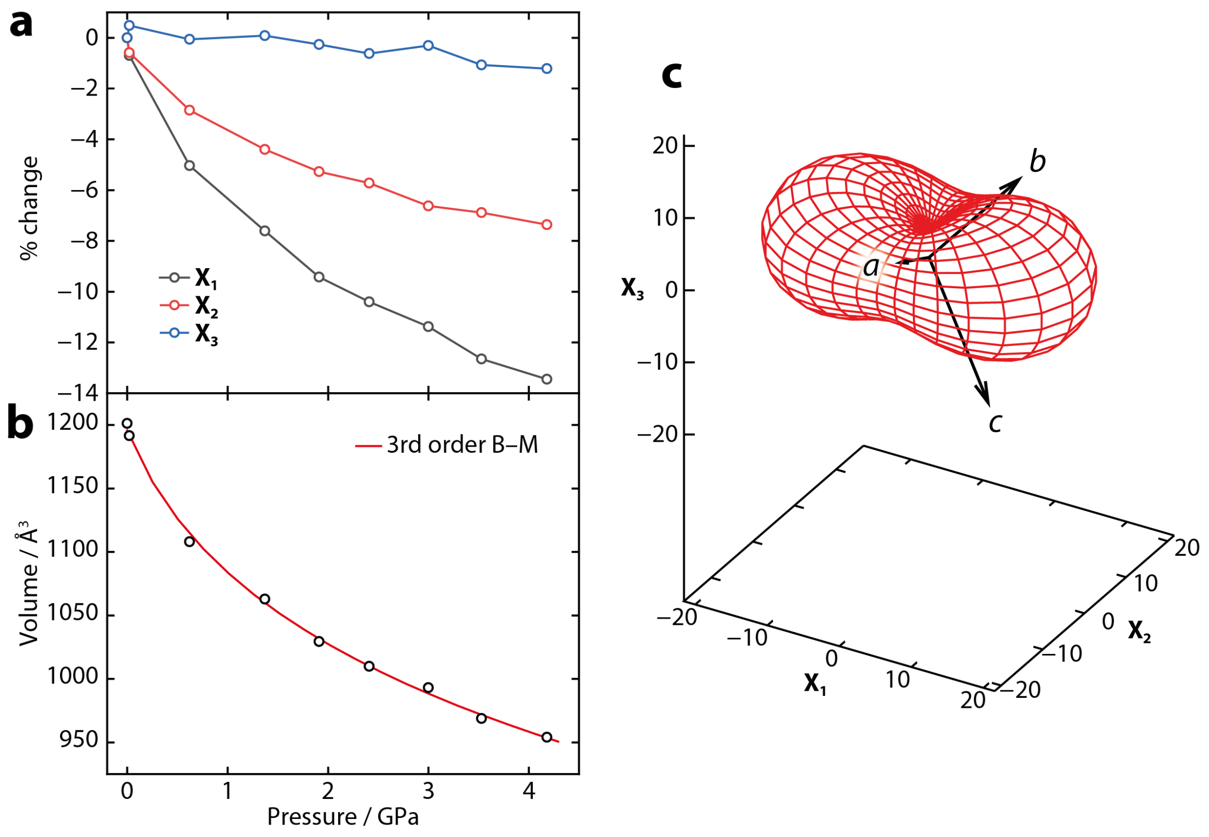

Figure S1: a) Percentage compression of principal (orthogonal) directions of the strain tensor. **b)** Cell volume as a function of pressure—the red line indicates the corresponding third-order Birch–Murnaghan equation of state. Error bars are within the size of the data points. **c)** Indicatrix (red mesh) illustrating the compressibility of each principal direction ($\mathbf{X_1}, \mathbf{X_2}, \mathbf{X_3}$), overlaid with the direct-space unit cell axes. The ambient pressure point used here was obtained on decompression following pressurisation of the sample.

3 High-pressure conformational change

Figure S2 shows plots of the conformational parameters, discussed in the main manuscript, for each pressure point. Neither of the dihedral angles show any convincing change as a function of pressure; though there are some approximate trends, the magnitude of the errors is such that most of the points are not significantly different from each other. The angle between mean planes, drawn through the phenyl and thiophene groups, and calculated using Mercury CSD,[S3] shows very little change ($< 1.0°$) over the full pressure range. An estimation of the errors is unavailable here, though the magnitude of the errors on the dihedral angles suggests that all the points measuring mean planes are almost certainly within error of each other.

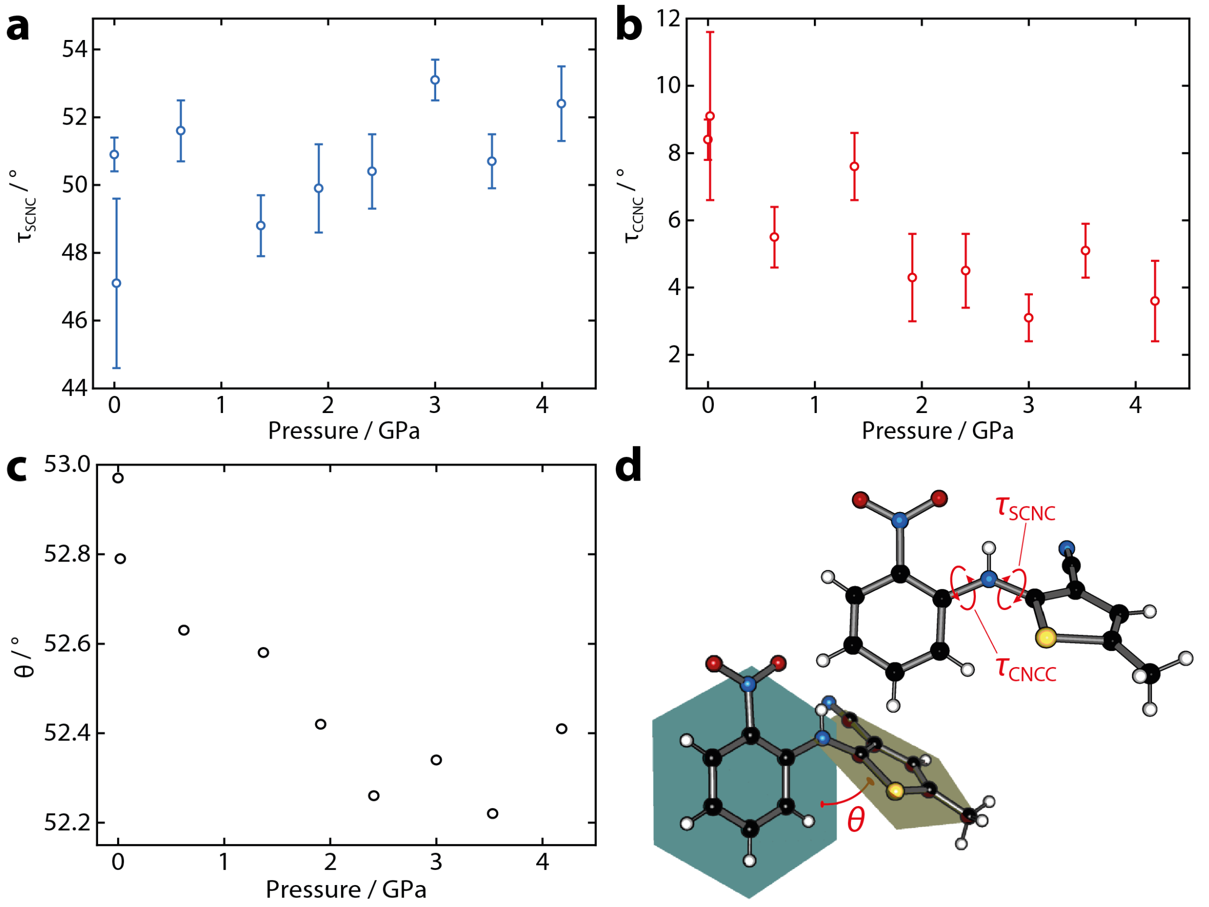

Figure S2: Dihedral angles for **a)** τ_{SCNC} and **b)** τ_{CCNC}; both y-axes are the same magnitude. **c** Change in angle between mean planes calculated through the phenyl and thiophene groups. **d)** A reproduction of Figure 1 showing a visual summary of the angles presented in panels **a–c**.

4 ROY—conformational potential energy surface

In the main manuscript we demonstrate the superior performance of the HSE06 hybrid density functional in calculating accurate electronic band gaps. Figure S3 presents the potential energy surface for a gas phase molecule of ROY as a function of τ_{SCNC}, calculated using this functional. The τ_{SCNC} angle was sampled in 15° intervals and held fixed at each value, while all other coordinates were allowed to relax. All the known polymorphs of ROY are marked on the plot with the position of their respective dihedral angles for reference, but because we optimise all other molecular geometry, there is no guarantee that our optimised structures necessarily resemble those of the known polymorphs. This is certainly the case for the R18 and R05 forms where there are two crystallographically-inequivalent molecules in the asymmetric unit.

We have not made to attempt to evaluate the relative polymorphs stabilities, as their Gibbs free energies are also dependent on temperature-related terms (vibrational enthalpy, entropy) that we do not consider here. Though these terms may be small, they will be highly–influential in the ROY family where there is only a very small energy separation between polymorphs.

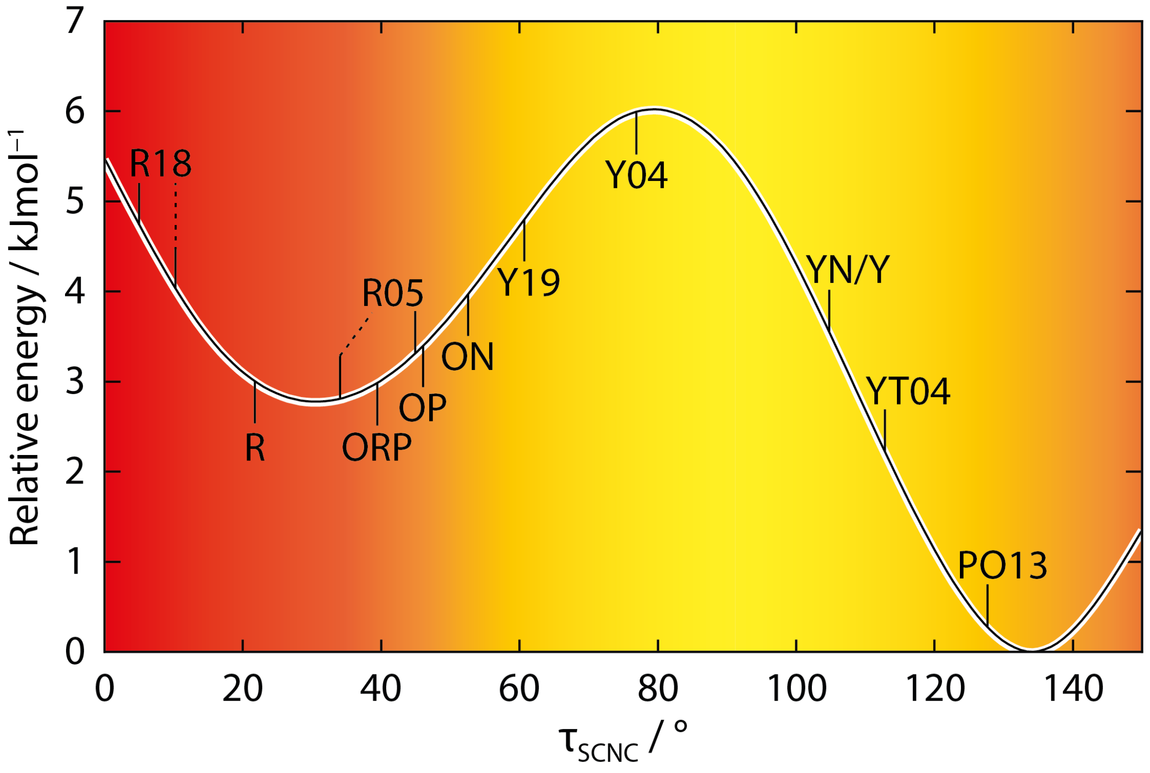

Figure S3: Potential energy surface as a function of τ_{SCNC}, calculated using the HSE hybrid density functional. The corresponding dihedral angle for each ROY polymorph is indicated. The R18 and R05 polymorphs both have two crystallographically inequivalent molecules in their asymmetric units, hence multiple entries here. The background colour is not quantitative and is simply provided as an approximate colour guide for the dihedral angles shown.

5 References

(S1) M. J. Cliffe, A. L. Goodwin, *J. Appl. Crystallogr.* **45**, 1321 (2012).

(S2) F. Birch, *Phys. Rev.* **71**, 809 (1947).

(S3) C. F. Macrae, *et al.*, *J. Appl. Crystallogr.* **53**, 226 (2020).
